# Photodynamic Activity of Protoporphyrin IX-Immobilized Cellulose Monolith for Nerve Tissue Regeneration

**DOI:** 10.3390/ijms23031035

**Published:** 2022-01-18

**Authors:** Ji Hye Lee, Ki Hong Kim, Oh Hyeong Kwon, Oh Kyoung Kwon, Hiroshi Uyama, Young-Jin Kim

**Affiliations:** 1 Department of Biomedical Engineering, Daegu Catholic University, Gyeongsan 38430, Korea; lllll_jh@naver.com; 2 Department of Optometry and Vision Science, Daegu Catholic University, Gyeongsan 38430, Korea; kkh2337@cu.ac.kr; 3 Department of Polymer Science and Engineering, Kumoh National Institute of Technology, Gumi 39177, Korea; ohkwon@kumoh.ac.kr; 4 Gastric Cancer Center, Kyoungpook National University Chilgok Hospital, Daegu 41404, Korea; kok007@hanmail.net; 5 Department of Applied Chemistry, Graduate School of Engineering, Osaka University, Suita 565-0871, Japan; 6 School of Advanced Materials and Chemical Engineering, Daegu Catholic University, Gyeongsan 38430, Korea

**Keywords:** photodynamic activity, reactive oxygen species, cellulose monolith, nerve regeneration, thermally induced phase separation

## Abstract

The development of nerve conduits with a three-dimensional porous structure has attracted great attention as they closely mimic the major features of the natural extracellular matrix of the nerve tissue. As low levels of reactive oxygen species (ROS) function as signaling molecules to promote cell proliferation and growth, this study aimed to fabricate protoporphyrin IX (PpIX)-immobilized cellulose (CEPP) monoliths as a means to both guide and stimulate nerve regeneration. CEPP monoliths can be fabricated via a simple thermally induced phase separation method and surface modification. The improved nerve tissue regeneration of CEPP monoliths was achieved by the activation of mitogen-activated protein kinases, such as extracellular signal-regulated kinases (ERKs). The resulting CEPP monoliths exhibited interconnected microporous structures and uniform morphology. The results of in vitro bioactivity assays demonstrated that the CEPP monoliths with under 0.54 ± 0.07 μmol/g PpIX exhibited enhanced photodynamic activity on Schwann cells via the generation of low levels of ROS. This photodynamic activation of the CEPP monoliths is a cell-safe process to stimulate cell proliferation without cytotoxic side effects. In addition, the protein expression of phospho-ERK increased considerably after the laser irradiation on the CEPP monoliths with low content of PpIX. Therefore, the CEPP monoliths have a potential application in nerve tissue regeneration as new nerve conduits.

## 1. Introduction

Peripheral nerve injuries, usually caused by traumatic lesions or tumor expiration, are always accompanied by dysfunction, movement and sensory disorders, and neuropathic pains, which seriously influence the patient’s quality of life [1,2]. Although peripheral nerves have an intrinsic potential to regenerate after lesions, damage involving extensive nerve tissue loss precludes the reconnection between proximal and distal nerve stumps [3]. Autologous transplantation is the main clinical intervention for repairing peripheral nerve injury. However, size mismatch, risk of neuroma formation, low graft availability, and permanent damage at the donating site extremely restrict the application of this technique [1,4].

Currently, nerve guidance conduits are considered a substitute for autografts [2]. These conduits should provide a favorable microenvironment for nerve regeneration while guiding axonal sprouting from the proximal to the distal stump. However, most of the commercially available tubular conduits provide an insufficient surface area to support the large number of cells needed to complete the functional regeneration of nerve tissue [5]. As one of the basic elements for nerve regeneration, the conduits play an important role in regulating cell growth and accelerating the formation of newborn nerve tissue through their three-dimensional porous structure [6]. Therefore, an ideal conduit for peripheral nerve regeneration should closely mimic the natural microenvironment of the peripheral nerve matrix.

Reactive oxygen species (ROS), such as hydrogen peroxide, superoxide anion, and hydroxyl radical, are generally small molecules that are short-lived and highly reactive [7,8]. The effect of ROS on cell fate depends on the level at which ROS are present. At low levels, ROS are considered essential for the regulation of normal physiological functions involved in development, such as cell proliferation and differentiation [9,10,11]. ROS also play an important role in the immune system and the maintenance of the redox balance. However, high levels of ROS cause detrimental oxidative stress that can lead to cell death by damaging proteins, nucleic acids, lipids, membranes, and organelles [12]. Therefore, modulating the generation of ROS is vital to cellular function.

Cellulose (CE)-based porous membranes with a three-dimensional (3D) interconnected pore structure, such as electrospun nanofibers, freeze-dried gels, aerogel monoliths, are one of the most useful solid materials in biomedical research [13,14,15,16]. These membranes have low density and a coherent network of loosely packed particles or nanofibrils [17]. They also exhibit other interesting characteristics, including high specific surface area and high porosity [14,18]. These attractive features make cellulose-based membranes a potential candidate for biomedical applications [13,14,15,16,17,18]. Various methods, including freeze-drying and thermally induced phase separation (TIPS), have been developed for fabricating cellulose-based porous membranes, especially cellulose monoliths [13,15,19]. The controllable and reproducible porous monoliths have been recognized as important materials for biomedical applications because their function depends on their pore and skeletal structures [20]. Therefore, the TIPS method has been generally utilized to fabricate cellulose monoliths due to its excellent versatility, simplicity, and controllability in determining morphology and pore structure, and is based on thermodynamically induced phase separation of polymer-rich and polymer-lean phases [15,19].

ROS are essential regulators of cell metabolism and are generated in virtually every cell type [21]. In this study, we hypothesized that low levels of ROS may stimulate nerve tissue regeneration. Therefore, we fabricated a protoporphyrin IX (PpIX)-immobilized CE (CEPP) monolith via simple TIPS and surface modification to promote nerve tissue regeneration through the activation of mitogen-activated protein kinases (MAPKs) involved in cell survival and proliferation. PpIX, a well-known photosensitizer, is produced in the body by the conversion of 5-aminolevulinic acid and acts as a ROS donor [22]. We systematically investigated the effects of polymer concentration on the pore and skeletal structures of the fabricated monoliths. To assess the applicability of the monoliths in nerve tissue regeneration, their physicochemical properties and bioactivity were evaluated.

## 2. Results and Discussion

### 2.1. Fabrication of the CEPP Monolith

**Figure 1:**
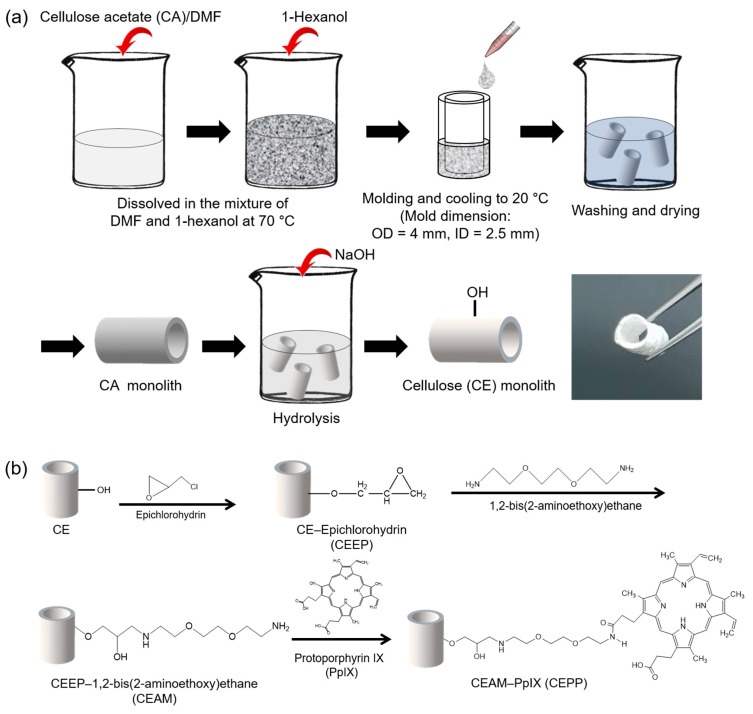
Schematic diagram of the fabrication of (**a**) CE and (**b**) CEPP monoliths.

A nerve conduit should ideally promote the proliferation of Schwann cells and fibroblasts in the nerve connective tissue to form Büngner bands for axonal regeneration [3]. For this purpose, the development of porous nerve conduits is an essential feature for the regeneration of nerve tissue as the pore structure prevents the infiltration of scar tissue while providing nutrients. In this study, the control CE monolith and PpIX-immobilized CE monoliths, namely CEPP10 (synthesized in 100 μg/mL PpIX solution), CEPP20 (synthesized in 200 μg/mL PpIX solution), and CEPP50 (synthesized in 500 μg/mL PpIX solution), were prepared using TIPS and surface modification, as shown in Figure 1. 

Various factors, such as solvent composition and polymer concentration, can affect the morphology and skeletal structure of the resulting monolith. Therefore, we first examined the effect of cellulose acetate (CA) concentration on the 3D pore structure of the CA monolith using dimethylformamide (DMF) as a good solvent and 1-hexanol as a poor solvent. These solvents controlled phase separation of CA in the fabrication of the monolith. Although all samples showed a 3D porous structure which was formed by phase separation, the pore and skeleton size in the CA monoliths gradually decreased with increasing CA concentration (Figure S1). The CA monoliths with lower concentrations of CA (6 and 8 *w*/*v*%) exhibited a higher bulk density and lower porosity (Figure S2). However, monoliths with higher concentrations (10 and 12 *w*/*v*%) had lower density and higher porosity. In addition, the pore and skeleton size in the CA monoliths also drastically decreased with an increase in CA concentration because of the slower phase separation and crystallization of CA due to the higher viscosity of the system [20]. The slower phase separation can slow down solvent transport from the CA-rich phase, leading to the formation of CA monoliths with decreased pore and skeleton size [23]. Therefore, the rate of phase separation and crystallization in the system dramatically affects the final morphology of the resulting CA monoliths. 

**Figure 2:**
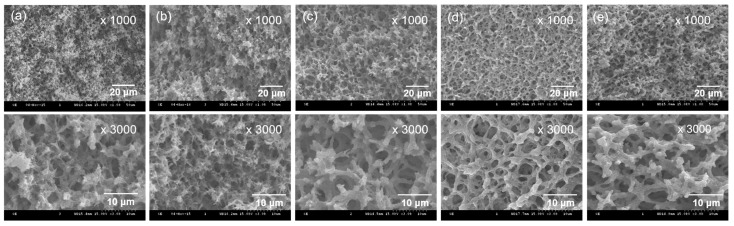
SEM images of (**a**) CA, (**b**) CE, (**c**) CEEP, (**d**) CEAM, and (**e**) CEPP (CEPP20) monoliths.

As a result of the fabrication of CA monoliths, the optimum CA concentration was established at 10 *w*/*v*% and thus was used in the following experiments to fabricate PpIX-immobilized CE monoliths. To prepare the CEPP monoliths, the control CE monolith was first generated by deacetylation of the CA monolith using a NaOH methanol solution at 25 °C (Figure 1). Next, epoxy and primary amine groups were introduced via the reaction of the CE monolith with epichlorohydrin and 1,2-bis(2-aminoethoxy) ethane to synthesize the epichlorohydrin-grafted CE (CEEP) and 1,2-bis(2-aminoethoxy) ethane-grafted CE (CEAM) monoliths, respectively. Finally, the resultant CEPP monoliths were prepared through the reaction of the CEAM monolith with PpIX. As shown in Figure 2, the morphology of the resulting monoliths was similar to that of CA monoliths. The porous morphology of the monoliths hardly changed after deacetylation and immobilization of PpIX, whereas the skeleton size was slightly increased. Moreover, the CEPP (CEPP10, CEPP20, and CEPP50) monoliths exhibited identical morphologies (data not shown). The amount of PpIX immobilized on the surface of the CE monolith was determined by fluorescence spectroscopy, which showed that the immobilized PpIX content was increased with increasing PpIX concentration in the reaction solution, which was 0.25 ± 0.03 μmol/g for CEPP10, 0.54 ± 0.07 μmol/g for CEPP20, and 0.99 ± 0.05 μmol/g for CEPP50.

### 2.2. Physicochemical Properties of the CEPP Monolith

**Figure 3:**
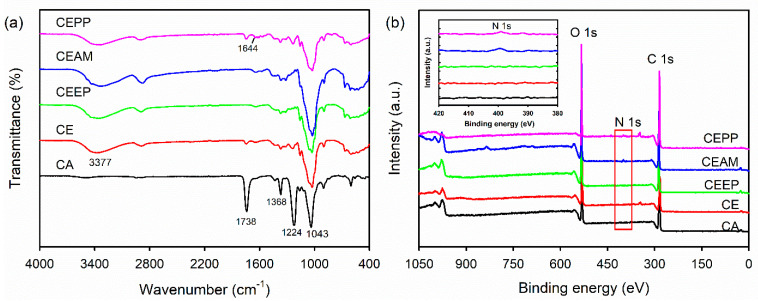
(**a**) ATR−FTIR and (**b**) XPS spectra of CA, CE, CEEP, CEAM, and CEPP (CEPP20) monoliths.

The change in the chemical structure during monolith surface modification in the fabrication of the CEPP monoliths was characterized using the attenuated total reflectance–Fourier transform infrared spectroscopy (ATR–FTIR). The characteristic absorption bands of the CA monolith were 1783, 1368, and 1224 cm^−1^, corresponding to the C=O, C–O, and C–CH_3_ stretching vibrations of the acetate groups (Figure 3a) [24]. In addition, the peak at 1043 cm^−1^ was due to the stretching vibration of the C–O–C groups present in the molecular chain of CA. The peaks corresponding to the acetate groups disappeared after deacetylation of the CA monolith, whereas the new absorption peak was centered at 3377 cm^−1^ due to the stretching vibration of the O–H groups in the CE. However, no differences in the characteristic bands were detected after surface modification of the CE monolith except a new peak at 1644 cm^−1^ appearing, corresponding to the C=O stretching vibration of amide bond after immobilization of PpIX. Moreover, the fabrication of the CEPP monolith was assessed through the determination of the surface elemental composition of the monoliths using X-ray photoelectron spectroscopy (XPS). As shown in Figure 3b, the CA, CE, and CEEP monoliths exhibited only two peaks because of the O 1 s orbital (533 eV) and the C 1 s orbital (285 eV). However, the CEAM and CEPP monoliths exhibited an additional peak corresponding to the N 1 s (399 eV) orbital of the primary amine.

### 2.3. In Vitro Bioactivity of the CEPP Monolith 

**Figure 4:**
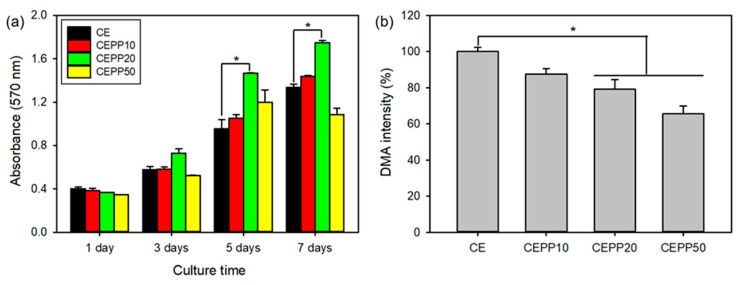
(**a**) Proliferation behavior of S16 cells cultured on the CEPP monoliths with laser irradiation (*n* = 5) and (**b**) fluorescence intensity of DMA due to generation of singlet oxygen by the CEPP monoliths (*n* = 6). The error bars indicate mean values ± SD. * *p* < 0.05 for the comparison between two treatment groups.

The in vitro bioactivity of CEPP monoliths was evaluated to determine their potential as conduits for guiding nerve tissue regeneration. This was done by investigating the proliferation of rat sciatic nerve Schwann cells (S16 cells) on the CEPP monoliths. The proliferation rate of S16 cells cultured on the CEPP monoliths was evaluated with the MTT assay in which higher cell viability translated as better monolith bioactivity. The number of attached S16 cells during the early stage was similar in all tested samples and a time-dependent increase in the number of cells on the monoliths was observed after 635 nm laser irradiation, as shown in Figure 4a. However, the proliferation rate of the cells was quite different, meaning that changes in the content of the immobilized PpIX on the CEPP monoliths significantly influenced the cell proliferation rate. After laser irradiation, the CEPP monoliths with a low content of PpIX (CEPP10 and CEPP20) effectively accelerated cell proliferation compared with the monolith with a high PpIX content (CEPP50). Among the CEPP monoliths, the CEPP20 monolith exhibited the fastest proliferation rate of the S16 cells at all tested time points. As these results were related to the PpIX content in the CEPP monoliths, there is an obvious CEPP-mediated photodynamic activity on Schwann cells via the generation of low levels of ROS.

The main action of photosensitizers is related to the production of ROS upon laser irradiation. PpIX is a well-known photosensitizer and effectively generates singlet oxygen (^1^O_2_) after laser irradiation. Therefore, to verify the enhanced bioactivity of the CEPP20 monolith, ^1^O_2_ generation from the CEPP monoliths during laser irradiation was quantitatively determined using 9,10-dimethylanthracene (DMA) as the ^1^O_2_ chemical probe. Fluorescent DMA reacts selectively with the ^1^O_2_ to form endoperoxide, causing a reduction in the fluorescence of DMA [25]. The ^1^O_2_ formation from the CEPP monoliths increased with increasing immobilized PpIX content on the monolith surface. As expected, the CEPP10 and CEPP20 monoliths exhibited low ROS generation, leading to enhanced bioactivity of the monoliths by promoting cell proliferation (Figure 4b). However, a high ROS generation was observed in the CEPP50 monolith, which considerably suppressed cell growth. Low levels of ROS are essential to regulate cell functions and to stimulate cell proliferation, acting as second messengers in cellular pathways [8]. In contrast, high levels of ROS induce detrimental oxidative stress that can lead to cell death. In addition, laser intensity also affected the proliferation rate of the S16 cells cultured on the CEPP20 monolith because higher laser intensity induces an increase in ROS generation (Figure S3) [26].

**Figure 5:**
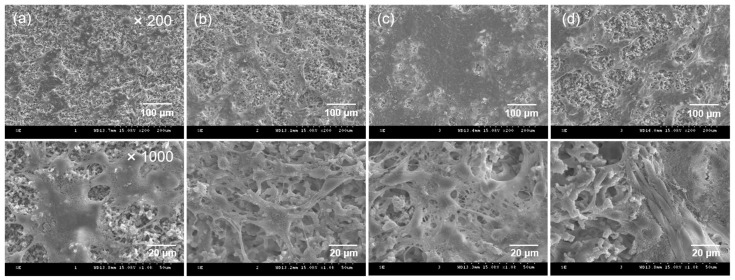
SEM images of cell growth on (**a**) CE, (**b**) CEPP10, (**c**) CEPP20, and (**d**) CEPP50 monoliths after culturing for 5 days plus laser irradiation.

The adhesion and morphology of S16 cells were observed using scanning electron microscopy (SEM) to examine cell proliferation on the CEPP monoliths after 5 days of culture with laser irradiation. SEM images showed that the S16 cells were successfully attached and had proliferated on the surface of the CEPP monoliths (Figure 5). The cells spread over almost the whole surface of the CEPP20 monolith after 5 days in culture plus laser irradiation. 

**Figure 6:**
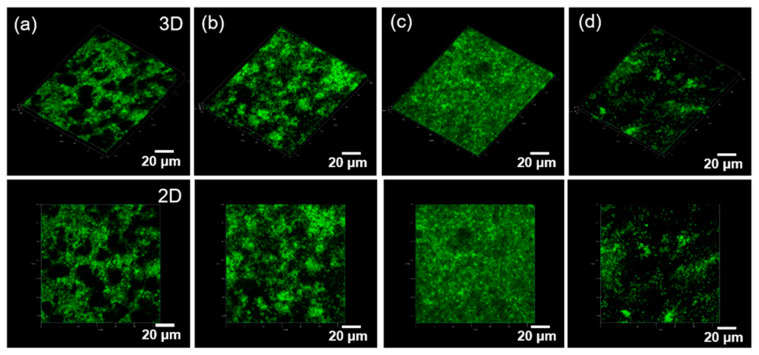
Fluorescence microscopy images of live cells stained with calcein-AM on (**a**) CE, (**b**) CEPP10, (**c**) CEPP20, and (**d**) CEPP50 monoliths after culturing for 5 days plus laser irradiation.

To visually evaluate the synergistic bioactivity of the CEPP monoliths, S16 cells were seeded on the CEPP monoliths and cultured for 5 days, and laser-irradiated. Then, cell proliferation was examined with a live-cell staining method and observed using a confocal laser scanning microscope (Figure 6). The viable cells showed green fluorescence upon staining with calcein-AM. The S16 cells had successfully proliferated on the surface of the CEPP monoliths after laser irradiation. The highest number of S16 cells was observed on the CEPP20 monolith. However, the number of stained cells on the CEPP50 monolith was lower than that observed on the other monoliths. These results were consistent with the MTT assay results. As a whole, these results indicate that the photodynamic activation of the CEPP monoliths with under 0.54 ± 0.07 μmol/g PpIX is a cell-safe process to stimulate cell proliferation without cytotoxic side effects.

**Figure 7:**
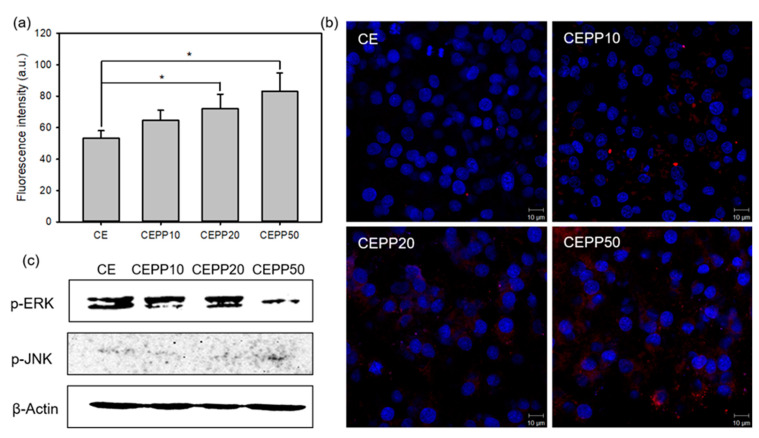
(**a**) DCF fluorescence intensity measured using a flow cytometer for determining the intracellular ROS level in the S16 cells culture on the CEPP monoliths after treatment with laser irradiation. The error bars indicate mean values ± SD (*n* = 5). * *p* < 0.05 for the comparison between the two treatment groups. (**b**) Confocal laser scanning microscopy images of the intracellular distribution of ROS in the S16 cells grown on the CEPP monoliths after being irradiated with a 635 nm laser. (**c**) Representative Western blots for phosphorylated ERKs and JNKs in lysates of S16 cells cultured on the CEPP monoliths after treatment with laser irradiation. β-Actin was used as the loading control.

ROS can induce significant cytotoxicity upon reaching an intracellular threshold concentration, leading to cell death through apoptosis or necrosis by irreversible biomolecular damage [27]. However, low levels of intracellular ROS promote cell proliferation. Therefore, the production amount of ROS is a very important factor for nerve tissue regeneration. The intracellular ROS generation in S16 cells after laser irradiation was determined with a flow cytometer using 2′,7′-dichlorodihydrofluorescein diacetate (DCF-DA) as a fluorescent indicator. DCF-DA can permeate live cells and is rapidly changed by ROS to green, fluorescent DCF [28]. DCF fluorescence intensity increased with increasing immobilized PpIX content on the monoliths, with the CEPP50 monolith exhibiting a significantly high fluorescence intensity compared with the fluorescence intensity of the other samples (Figure 7a). This was due to the elevated ROS production from the CEPP50 monolith, which led to high cytotoxicity for the S16 cells. This result was confirmed by observing the cells using a confocal laser scanning microscope. Red fluorescence signals due to intracellular ROS generation were observed in S16 cells that were cultured on the surface of the CEPP monoliths and laser-irradiated. As shown in Figure 7b, these signals increased with increasing immobilized PpIX content on the monoliths.

Moreover, the protein expression associated with cell proliferation and death in the S16 cells was monitored by Western blot analysis. The MAPK family, including c-Jun N-terminal kinases (JNKs) and extracellular signal-regulated kinases (ERKs), has a fundamental role in both cell survival and the induction of cell death [29,30]. ERKs are mainly activated by growth factors, which can stimulate cell proliferation. However, JNKs mediate cellular responses to stress and may lead to cell death. The protein expression of phospho-ERK (p-ERK) decreased considerably after laser irradiation of the CEPP50 monolith, whereas the expression of phospho-JNK (p-JNK) increased (Figure 7c). In particular, the S16 cells cultured on the CEPP20 monolith exhibited higher p-ERK expression and lower p-JNK expression than those on other monoliths. Taken together, these results suggest that the CEPP20 monolith may be an effective nerve conduit for guiding nerve tissue regeneration.

## 3. Materials and Methods

### 3.1. Materials

Cellulose acetate (CA, Mn = 50,000), epichlorohydrine, 1,2-bis(2-aminoethoxy) ethane, N-(3-dimethylaminopropyl)-N′-ethylcarbodiimide hydrochloride (EDC), N-hydroxysuccinimide (NHS), 3-(4,5-dimethyl-2-thiazolyl)-2,5-diphenyl-2H-tetrazolium bromide (MTT), bisBenzimide H 33258 (Hoechst 33258, H-33258), dimethylsulfoxide (DMSO), DMF, and 1-hexanol were obtained from Sigma–Aldrich (St. Louis, MO, USA). Protoporphyrin IX (PpIX) was purchased from Frontier Scientific (Logan, UT, USA). A rat sciatic nerve Schwann cell line (S16) was purchased from the Korean Cell Line Bank (KCLB, Seoul, Korea). Dulbecco’s modified Eagle’s medium (DMEM), fetal bovine serum (FBS), penicillin–streptomycin, and Dulbecco’s phosphate-buffered saline (DPBS, pH 7.4) were obtained from Gibco BRL (Waltham, MA, USA). LIVE/DEAD Viability/Cytotoxicity Assay Kit, CellROX™ Deep Red Reagent, and 2′,7′-dichlorodihydrofluorescein diacetate (DCF-DA) were obtained from Molecular Probes (Eugene, OR, USA). PRO-PREP™ Protein Extraction Solution was purchased from iNtRon Biotechnology (Sungnam, Korea) and the BCA Protein Assay Kit was purchased from ThermoFisher Scientific (Waltham, MA, USA). Anti-phospho ERK, anti-phospho JNK, and anti-β-actin antibodies were purchased from Cell Signaling Technology (Danvers, MA, USA). ECL™ Western Blotting Detection Reagents were obtained from GE Healthcare (Little Chalfont, England). Other reagents were commercially purchased and were used as received.

### 3.2. Fabrication of the CE Monolith

The CE monolith was prepared through the TIPS method and CA deacetylation. Briefly, CA (2 g) was first dissolved in 8 mL of a good solvent DMF, followed by the dropwise addition of 12 mL of a poor solvent, 1-hexanol, at 70 °C for 1 h until the mixture became transparent. We varied the concentration of CA as 6, 8, 10, and 12 *w*/*v*%. The obtained solution was injected into a polytetrafluoroethylene (PTFE) mold with a 4 mm cylindrical void and then a 2.5 mm PTFE stick was inserted into the mold to create the cavity of the nerve conduit. The mold was cooled to 20 °C and maintained at that temperature for 24 h for the TIPS process. The CA monolith was fabricated and repeatedly washed with methanol before being dried under vacuum. The CE monolith was then produced by the deacetylation of the CA monolith with 0.5 N NaOH methanol solution at 25 °C for 3 h. After neutralization with 0.5 N HCl, the CE monolith was washed with distilled water (DW) and then vacuum dried.

### 3.3. Preparation of the PpIX-Immobilized CE Monolith

The CE monolith (0.5 g) was immersed in 25 mL of 1 N NaOH solution at 50 °C for 1 h. Epichlorohydrin (4.5 mL) was then added to the mixture, and the reaction was continued to synthesize the CEEP monolith at 50 °C for 2 h. The CEEP monolith was washed thoroughly with DW until the eluate became neutral and then dried in vacuo. Subsequently, the CEEP monolith (0.1 g) was immersed into a 7.5 mL ethanol solution containing 1,2-bis(2-aminoethoxy) ethane (0.7 g) at 50 °C for 24 h, followed by washing and drying to fabricate the CEAM monolith. 

To prepare the PpIX-immobilized CE (CEPP) monolith, PpIX (1, 2, or 5 mg), used as a ROS donor, was dissolved in 10 mL DMSO containing EDC (25.9 mg) and NHS (15.6 mg) before immersing the CEAM monolith into the DMSO solution. The final concentration of PpIX in the solution was 100 (CEPP10), 200 (CEPP20), or 500 μg/mL (CEPP50). The reaction was allowed to proceed for 12 h at 40 °C under gentle shaking. The resultant CEPP monolith was rinsed repeatedly with DW and dried in vacuo. The amount of immobilized PpIX on the surface of the CEPP monolith was quantified by measuring the fluorescence spectra of PpIX (excitation, 405 nm; emission, 635 nm) using a spectrofluorophotometer (LS55, Perkin–Elmer, Waltham, MA, USA) as follows. A known amount of PpIX was dissolved in DMSO and the fluorescence intensity was measured using a spectrofluorophotometer to prepare a calibration curve. The concentration of PpIX immobilized on the CEPP monoliths was calculated from the change in fluorescence intensity of reactant solution before and after the immobilization of PpIX.

### 3.4. Surface Morphology and Physicochemical Properties of the PpIX-Immobilized CE Monolith

The morphologies of CEPP monoliths were observed with SEM (S-4300, Hitachi, Tokyo, Japan) after sputter-coating samples with gold. The chemical structures of the monoliths were analyzed by FTIR (ALPHA spectrometer, Bruker Optics, Billerica, MA, USA) in ATR mode accessorizing with a monolithic diamond crystal. FTIR spectra were collected in the wavenumber range from 400 to 4000 cm^−1^ with a resolution of 4 cm^−1^ and 24 scans. The surface elemental composition of the monoliths was determined via XPS analysis using a Quantera SXM (ULVAC-PHI Inc., Kanagawa, Japan) equipped with a monochromatic Al Kα X-ray source (1486 eV). The density of monoliths was determined using their geometric dimensions and weight values. A mercury porosimeter (AutoPore IV 9520, Micromeritrics Instrument Corp., Norcross, GA, USA) was used to measure the porosity of monoliths. The porosity was defined as the volume of the pores divided by the total volume of monoliths.

### 3.5. Singlet Oxygen Detection

^1^O_2_ generation from the CEPP monoliths under different conditions was detected using DMA as the ^1^O_2_ chemical probe. Eighty milligram of CEPP monolith was immersed into a 12-well tissue culture plate filled with 2 mL of DMA solution (20 μM in DMF), followed by a 1 min irradiation at a light power density of 30 mW/cm^2^ using a 635 nm laser source. The fluorescence spectra of DMA (excitation, 360 nm; emission, 430 nm) as a result of the photosensitization reaction were monitored with a Perkin–Elmer LS55 spectrofluorophotometer (Waltham, MA, USA).

### 3.6. In Vitro Bioactivity of the PpIX-Immobilized CE Monolith 

Synergistic bioactivity mediated by low levels of ROS in the CEPP monoliths was investigated by MTT assay. All cultures were incubated in DMEM supplement with 10% FBS and 1% penicillin–streptomycin at 37 °C with 5% CO_2_. Prior to cell seeding, the CEPP monoliths were sterilized with a graded series of ethanol (75%, 50%, and 25%) and 3 h of UV irradiation, then rinsed five times each with DPBS and DMEM. These monoliths were then placed into a 48-well tissue culture plate. S16 cells (5 × 10^4^ cells per well) were seeded onto the sterilized monoliths in DMEM containing 10% FBS and incubated at 37 °C for 24 h. The bioactivity of the CEPP monoliths was evaluated by exposing the cells to 635 nm irradiation (30 mW/cm^2^, 1 min) and the cells were then incubated for another 4 h. The irradiation was performed with a 635 nm laser every 48 h and the viability of irradiated cells was measured spectrophotometrically at 570 nm according to the MTT protocol at each time point. Under the same conditions of the MTT assay, cell attachment of S16 cells cultured on the CEPP monoliths was observed with SEM. S16 cells incubated on the monoliths were fixed with 4% glutaraldehyde for 1 h and then rinsed three times using DPBS. After dehydrating with a graded series of ethanol, the samples were vacuum dried, and gold-coated for SEM analysis.

S16 cell viability after laser irradiation was visualized using the LIVE/DEAD Viability/Cytotoxicity Assay Kit, in which calcein-AM stains live cells green [31]. The S16 cells (5 × 10^4^ cells per well) were seeded onto the monoliths and cultured at 37 °C for 24 h and then were irradiated with a 635 nm laser (30 mW/cm^2^, 1 min). Next, the cells were irradiated twice with a 635 nm laser every 48 h and were stained for 30 min at room temperature with 1 μM of calcein-AM. This was followed by rinsing with DPBS and incubation for another 24 h. Live cells were visualized using an inverted LSM 700 confocal laser scanning microscope (Carl Zeiss, Oberkochen, Germany).

### 3.7. Intracellular ROS Generation Assays

The intracellular ROS generation was quantitatively determined using a flow cytometer. The S16 cells (5 × 10^4^ cells per well) were seeded onto the CEPP monoliths and cultured at 37 °C for 24 h, followed by the 635 nm laser irradiation (30 mW/cm^2^, 1 min). The irradiation and incubation were performed three times every 48 h; afterward, the cells were treated with DCF-DA at 37 °C for 30 min. Lastly, the ROS concentration was measured using a flow cytometer (excitation, 488 nm; emission, 530 nm). The intracellular ROS generation was also observed using confocal laser scanning microscopy after treating the cells with CellROX™ Deep Red Reagent and H-33258.

### 3.8. Western Blot

The S16 cells (5 × 10^5^ cells per well) were seeded onto the monoliths, cultured at 37 °C for 24 h, and irradiated twice with a 635 nm laser (30 mW/cm^2^, 1 min) every 24 h. After incubation for another 24 h, the S16 cells were rinsed three times with DPBS and lysed in a PRO-PREP™ Protein Extraction Solution containing a 1× protease and phosphatase inhibitor cocktail. The concentration of proteins was determined using a BCA Protein Assay Kit. Lysates (10–30 μg protein) were isolated by 10% sodium dodecyl sulfate–polyacrylamide gel electrophoresis and the resolved proteins were transferred to polyvinylidene fluoride (PVDF) membranes blocked in DPBS with Tween 20 and 5% skim milk for 1 h at 25 °C. The PVDF membranes were incubated with primary antibodies and a secondary horseradish peroxidase-conjugated antibody, after which the immunoreactive bands were observed by chemiluminescence detection. In addition, the β-actin antibody was used as a control to confirm equal loading of the proteins. Data were analyzed using the Davinch-Chemi CAS-400SM Western Imaging System (Davinch-K, Seoul, Korea).

### 3.9. Statistical Analysis

The data are expressed as the mean value ± standard deviation (SD) from at least four independent experiments. Two-group parameters were analyzed using one-way analysis of variance, followed by Tukey’s test with the SigmaPlot 13.0 software (Systat Software Inc., San Jose, CA, USA). Significance was set at * *p* < 0.05.

## 4. Conclusions

In this study, simple TIPS and surface modification were employed to produce the CEPP monoliths for promoting nerve tissue regeneration through the activation of MAPKs involved in cell survival and proliferation. The resulting monoliths exhibited interconnected microporous structures. The immobilized PpIX content influenced the in vitro bioactivity of the CEPP monoliths. Low levels of ROS generated from the CEPP monoliths improved the photodynamic activity of the monoliths on Schwann cells without severe cytotoxic side effects due to the activation of ERKs and the suppression of JNKs. Based on these results, our simple method for fabricating CEPP monoliths may contribute to the development of novel nerve conduits for nerve tissue regeneration.

## Data Availability

The data presented in this study are available on request from the corresponding author.

## Supplementary Materials

The following are available online at https://www.mdpi.com/article/10.3390/ijms23031035/s1.
