# Supplementary material for: Photodynamic Activity of Protoporphyrin IX-Immobilized Cellulose Monolith for Nerve Tissue Regeneration
Source: Int J Mol Sci. 2022 Jan 18;23(3):1035. doi: 10.3390/ijms23031035 (PMC8835291; doi:10.3390/ijms23031035)
Supplement: Supplementary file 1 [file ijms-23-01035-s001.zip › ijms-1552242-supplementary.pdf]

Supplementary Information

Photodynamic Activity of Protoporphyrin IX-Immobilized Cellulose Monolith for Nerve Tissue Regeneration

Ji Hye Lee, Ki Hong Kim, Oh Hyeong Kwon, Oh Kyoung Kwon, Hiroshi Uyama*, Young-Jin Kim*

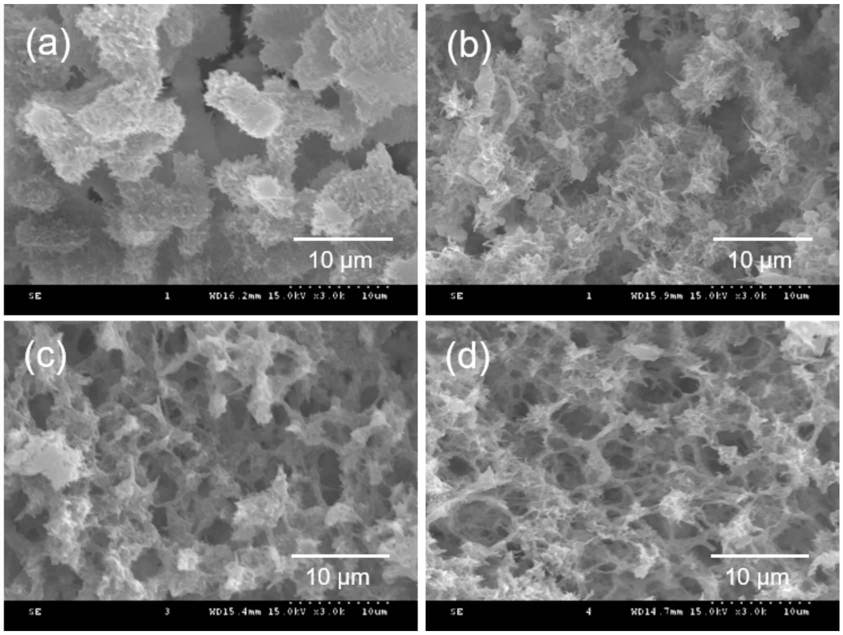

Figure S1. SEM images of CA monoliths fabricated with various CA concentrations; (a) 6 w/v%, (b) 8 w/v%, (c) 10 w/v%, and (d) 12 w/v%.

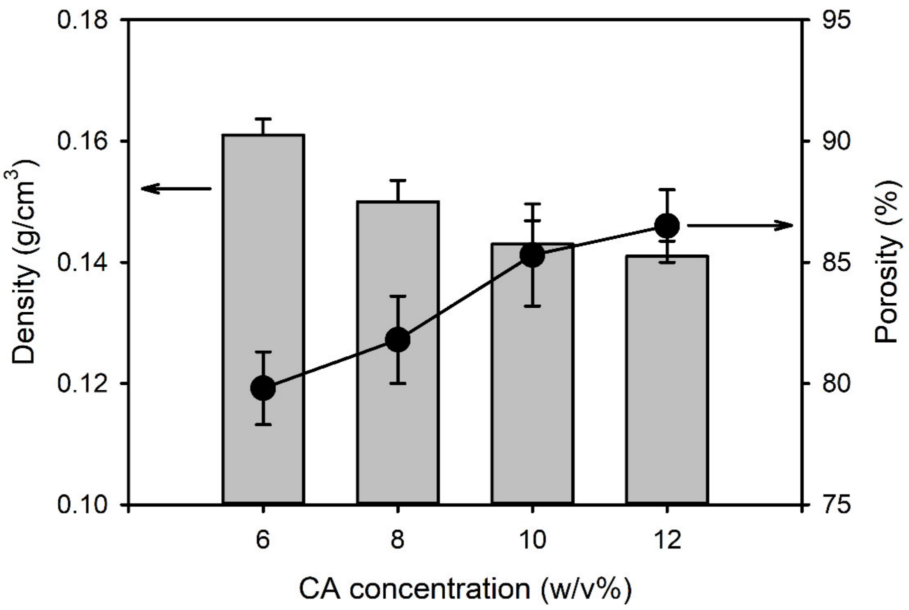

Figure S2. Density and porosity of CA monoliths with different CA concentrations.

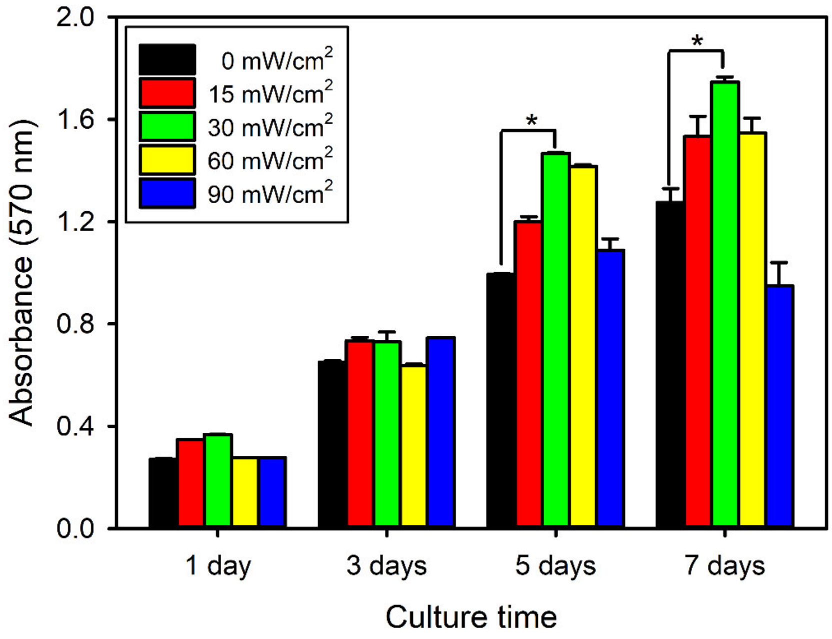

Figure S3. Proliferation behaviors of S16 cells cultured on the CEPP20 monolith upon laser irradiation with different laser intensity (*n* = 5). $p^* < 0.05$ for the comparison between two treatment groups.
